# Draft Genome Sequence of *Aeromonas molluscorum* Strain 848T^T^, Isolated from Bivalve Molluscs

**DOI:** 10.1128/genomeA.00382-13

**Published:** 2013-06-20

**Authors:** Nino Spataro, Maribel Farfán, Vicenta Albarral, Ariadna Sanglas, J. Gaspar Lorén, M. Carmen Fusté, Elena Bosch

**Affiliations:** Institute of Evolutionary Biology (CSIC-UPF), Department of Experimental and Health Sciences, Universitat Pompeu Fabra, Barcelona, Spaina; Departament de Microbiologia i Parasitologia Sanitàries, Facultat de Farmàcia, Universitat de Barcelona, Barcelona, Spainb; Institut de Recerca de la Biodiversitat (IRBio), Universitat de Barcelona. Barcelona, Spainc

## Abstract

We report here the draft genome sequence of *Aeromonas molluscorum* 848T, the type strain of this *Aeromonas* species, which was isolated from wedge shells (*Donax trunculus*) obtained from a retail market in Barcelona, Spain, in 1997.

## GENOME ANNOUNCEMENT

The genus *Aeromonas* Stanier 1943, 213^AL^, comprises a collection of Gram-negative, rod-shaped, non-spore-forming, oxidase- and catalase-positive, glucose-fermenting, facultatively anaerobic bacteria that are resistant to vibriostatic agent O/129 and generally motile by means of polar flagella (1). The genus *Aeromonas* belongs to the family *Aeromonadaceae* within the *Gammaproteobacteria*. Aeromonads are autochthonous to aquatic environments worldwide and are usual microbiota (as well as primary or secondary pathogens) of fish, amphibians, and other animals. Some motile species (mainly *Aeromonas caviae*, *Aeromonas hydrophila*, and *Aeromonas veronii* bv. Sobria) are opportunistic pathogens of humans (2). *Aeromonas molluscorum* was defined on the basis of a group of five strains that were isolated from bivalve molluscs obtained from retail markets in Barcelona in 1997 (3), which clustered together as a separate phenon in a phenotypic study (4). The type strain of this new *Aeromonas* species is *A. molluscorum* 848T (CECT 5864^T^, LMG 22214^T^). Recently, in 2010, a new strain of *A. molluscorum* was isolated from a tributyltin (TBT)-contaminated sediment in Ria de Aveiro, Portugal (5).

The draft genome sequence of the *A. molluscorum* type strain was obtained with a shotgun strategy using Roche 454 sequencing technology. A total of 122,746 reads with an average length of 404 nucleotides (9× coverage) were *de novo* assembled using a combined strategy (Newbler *de novo* and Velvet *de novo*). A total of 309 contigs, 304 of >1 kb in length, were constructed, with an *N*_50_ of 21,565 bp; the largest contig assembled measured 138,647 bp and the calculated genome size was 4.24 Mb, which is slightly smaller than the other *Aeromonas* genomes reported to date (ranging from 4.3 to 4.97 Mb) (6–14). The G+C mole percentage was 59.2.

The gene prediction and protein annotation were performed by applying the NCBI Prokaryotic Genomes Automatic Annotation Pipeline (http://www.ncbi.nlm.nih.gov/genomes/static/Pipeline.html) on the contigs assembled. A total of 3,946 protein-coding sequences were identified, together with 70 tRNA genes and 3 rRNA genes. Protein annotation using the VFDB database (http://www.mgc.ac.cn/VFs/) of virulence factors for bacterial pathogens detected six putative virulence factors, including a gene involved in ferric uptake (*hemE*), a gene encoding a phosphoheptose isomerase associated with lipopolysaccharide (LPS) biosynthesis (*gmhA*), two genes (*cheW* and *cheY*) involved in signal transmission to the flagellar motor switch component, and a regulator gene responsible for epithelial cell invasion (*csrA*). We detected a 36.4-kb putative prophage showing high identity to *phi*O18P, a bacteriophage isolated from *A. media* (15) and also detected in the genome sequence of *A. caviae* Ae398 (8). In addition to the complete sequence of *phi*O18P, 3 partial bacteriophage sequences were also detected. Two copies of an insertion sequence (IS) with high homology to ISAsa4 (88% identity) were discovered using the server ISFinder (http://www-is.biotoul.fr//). This IS element was previously reported in atypical strains of *A. salmonicida* subsp. *salmonicida* (16).

### Nucleotide sequence accession numbers.

This Whole-Genome Shotgun project has been deposited at DDBJ/EMBL/GenBank under the accession number AQGQ00000000 (BioProject PRJNA183610). The version described in this paper is the first version, AQGQ01000000.
